# Quality of Life of Children with Cerebral Palsy and Its Association with Their Physical Activity Levels: A Cross-Sectional Study

**DOI:** 10.3390/healthcare13172166

**Published:** 2025-08-30

**Authors:** Reem A. Albesher, Reem M. Basoudan, Areej Ghufayri, Dana Aldayel, Dareen Fagihi, Shahad Alzeer, Shaima Althurwi, Nouf Aljarallah, Turki Aljuhani, Mshari Alghadier

**Affiliations:** 1 Department of Rehabilitation Sciences, College of Health and Rehabilitation Sciences, Princess Nourah bint Abdulrahman University, Riyadh 11671, Saudi Arabia; raalbesher@pnu.edu.sa (R.A.A.);; 2 Department of Occupational Therapy, College of Applied Medical Sciences, King Saud Bin Abdulaziz University for Health Sciences, Riyadh 11481, Saudi Arabia; juhanit@ksau-hs.edu.sa; 3 King Abdullah International Medical Research Center, Riyadh 11481, Saudi Arabia; 4 Department of Health and Rehabilitation Sciences, Prince Sattam Bin Abdulaziz University, Alkharj 11942, Saudi Arabia; m.alghadier@psau.edu.sa

**Keywords:** cerebral palsy, quality of life, physical activity, school-age children

## Abstract

Background/Objectives: Children, caregivers, and physicians may be insufficiently aware of the effect(s) of physical activity levels on the quality of life (QoL) of children with cerebral palsy (CP). This study aimed to understand the levels of physical activity of school-age children with CP compared with typically developing (TD) peers, and to examine the relationship between physical activity levels and the QoL of children with CP. Methods: We conducted a cross-sectional study of children with CP and TD children aged 6–12 years. Parents of children with CP completed a four-section survey: demographic information, parent-reported Gross Motor Functional Classification System, physical activity, and the CP-QoL questionnaire. Parents of TD children completed the demographic and physical activity sections. To account for the severity of motor impairment associated with CP, further analysis was conducted to compare QoL between the ambulant and non-ambulant groups of children with CP. Results: Eighty-two participants were included in the analysis: 42 children with CP and 40 TD children (8.29 ± 1.79 years; 8.35 ± 1.76 years). The lowest QoL domain scores were access to service, pain, and effect(s) of disability. Children with CP reported similar physical activity levels to those of the TD children. Physical activity levels were associated with the general QoL score, and feeling-social domains of QoL. Conclusion: Our findings support the positive prediction of high physical activity levels with QoL among school-aged children with CP.

## 1. Introduction

Cerebral palsy (CP) is an umbrella term that describes disorders affecting movement and posture development and limiting activities due to non-progressive disruptions to the fetal or infant brain [1]. It can affect various aspects of the child’s life, including motor and cognitive abilities. CP is one of the most common causes of childhood physical disability [2]. The wide variation in its clinical presentation is a key feature of CP [1]. The diagnosis of CP encompasses a range of abnormalities that impair posture and mobility, resulting in limitations in daily activities [1]. Additionally, individuals with CP may experience seizure disorders and changes in sensation, cognition, communication, perception, and behavior [1]. Studies have shown that children with CP generally exhibit lower physical activity levels than their typically developing (TD) peers [3]. Hence, there is an increased interest in the degree to which children with CP engage in physical activity [4]. The Gross Motor Function Classification System (GMFCS) is the gold standard when classifying the severity and functional level in children with CP [5].

The World Health Organization (WHO) defines quality of life (QoL) as “the individual’s perception of their position in life in the context of the culture and value system in which they live, and in relation to their goals, expectations, standards, and concerns” [6]. Generally recognized as a multidimensional subjective concept, QoL can be assessed using self-reported measures that are either generic or condition-specific [7]. The accurate disease-specific tool to measure the QoL of children with cerebral palsy (CP) is the CP-QOL [8]. Disease-specific QoL tools generally include items and domains that reflect the lived experiences of children and adolescents with CP, such as their feelings about disability [8]. Obtaining reliable information about the QoL of children with severe intellectual impairments or significant communication challenges can be difficult. In such cases, proxy measures of QoL, often through parental reports, are necessary [8]. Although such reports may represent a different perspective from that of the child, they play an important role in assessing the QoL of children with CP [8]. Parental perspective is essential in family-based approaches to CP rehabilitation because parents can provide valuable insights into their child’s life when direct self-reporting is not feasible [7,9]. This focus allows parents to provide a more accurate assessment of the QoL of their children [8]. There is growing evidence that there is a correlation between physical activity and the QoL of children with CP, although many studies have used non-specific QoL measures [10].

Functional ability, as measured by the GMFCS, is one of the factors influencing various QoL measures of physical domains [11]. However, no apparent relationship exists between functional ability and psychological domains [11]. Furthermore, pain has adverse effects on QoL across various domains [12]. According to the WHO, physical activity is defined as any movement driven by skeletal muscles that involves energy expenditure, encompassing both leisure activities and commuting [13]. While we do not have CP-specific physical activity recommendations, the WHO recommends that children and young adults aged 5 to 17 engage in moderate-to-vigorous physical activity, primarily in the form of aerobic exercise, for at least 60 min a day, on average, throughout the week [13]. This activity should include at least three days of vigorous aerobic activity combined with exercises that strengthen muscles and bone mass [13].

Research is needed to understand the association between the physical activity levels and the QoL of children with CP. In particular, this association is yet to be investigated in Saudi Arabia, where cultural and contextual factors may play a significant role. Current evidence on the association between physical activity and QoL in children with CP in Saudi Arabia is limited to non-diagnosis-specific tools [14], environmental QoL [15] or measure caregiver’s QoL [16]. The present study aimed to (1) assess levels of physical activity among school-age children with CP compared with TD peers and (2) examine the relationship between levels of physical activity and QoL among school-age children with CP. We hypothesized that a positive association would be found between the physical activity levels and the QoL of school-age children with CP.

## 2. Materials and Methods

### 2.1. Study Design and Participants

A cross-sectional design, based on a caregiver-reported online questionnaire, was used to assess the levels of physical activity and QoL of children with CP. Quantitative data were collected between 10 March and 20 April 2024, using online surveys. Caregivers of children with CP were recruited based on their availability and willingness to participate in the study. The sample for this study consisted of parents or caregivers of children between 6 and 12 years old with varying subtypes and severity levels of CP. A normative control group of TD children in the same age range was also recruited. Children with CP were recruited from rehabilitation centers/hospitals during regular medical and therapy visits. The typically developing group was recruited from schools and community centers. The exclusion criteria were children less than 6 years of age, those over 12 years of age, and caregivers who did not read Arabic. The exclusion criteria for typically developing children were severe pain that interfered with activity or the presence of a mental or physical disability. Ethical approval was granted by the Institutional Review Board at Princess Nourah bint Abdulrahman University, Riyadh, Kingdom of Saudi Arabia (Approval No. H-01-R-059), in accordance with the Helsinki Declaration of Ethical Principles. Informed consent was obtained from the parents or caregivers of the participating children. The data presented in this study are available on request from the corresponding author.

### 2.2. The Survey Instrument

The instrument was divided into four main sections: Demographic information, Parent-reported Gross Motor Functional Classification System (GMFCS), Physical Activity, and the CP QoL-Questionnaire. Demographic information included the child’s age, gender, school attendance, relationship to the respondent, and educational level.

#### 2.2.1. Cerebral Palsy Classification System

The GMFCS is a classification system used to assess the motor skills of children with CP from infancy to 18 years of age [17]. It categorizes children into five levels based on their ability to sit, walk, and exercise on mobile devices [17]. The GMFCS levels range from GMFCS I, the least impaired level, to GMFCS V, representing the most impaired level [5]. This classification system helps determine the level of support that children with CP need to perform different motor skills in a variety of situations [18]. The Arabic GMFCS-Extended and Revised (E&R) is a reliable and user-friendly classification system for use by parents and caregivers of children with CP [19]. In this study, the GMFCS level was determined by parents using the appropriate age version of the Arabic Family Report Questionnaires, specifically the GMFCS Family & Self Report Questionnaire in Arabic [20]. The children in the CP group were classified into two groups based on their GMFCS levels: Ambulant GMFCS I–III and Non-Ambulant GMFCS IV–V.

#### 2.2.2. Cerebral Palsy Quality of Life Questionnaire-Child

The CP QoL Questionnaire for Children, also known as the CP QoL-Child, is a questionnaire designed to measure the QoL of children aged 4–12 years with CP [21]. The Arabic version of the primary caregiver CP-QoL-Child was used in this study. The primary caregiver QoL-Child version is designed for children between 4 and 12 years, and includes 66 items in seven domains (Social well-being and Acceptance, Functioning, Participation and Physical Health, Emotional Well-being, Pain and Impact of Disability, Access to Services, and Family Health [21]. The CP QoL-Child items focus on assessing well-being and avoiding the inclusion of items that could negatively affect the individual’s self-esteem [22].

#### 2.2.3. Parent-Reported Physical Activity

This part of the survey included five main questions about the child’s frequency, intensity, duration, type, and location of physical activities. The survey questions were adopted from a valid and reliable measure developed by Prochaska et al. (2001) [23]. Physical activity questions were translated into Arabic using a forward–backward translation process by a certified translation center to ensure the validity of the Arabic version. Definitions of different types of physical activity were provided to ensure accurate reporting by caregivers. The Physical activity questions were as follows:

1. Physical activities levels: Over the past 7 days, on how many days were you physically active for a total of at least 60 min per day? With physical activity we mean any activity that increases your heart rate and makes you get out of breath some of the time. Response options: 0 days, 1 day, 2 days, 3 days, 4 days, 5 days, 6 days to 7 days.
2. Days of moderate to vigorous physical activities: How many days did your child practice physical activities to strengthen his/her bones and muscles (e.g., rope jumping, climbing an elevated object, running)? Response options: 0 days, 1 day, 2 days, 3 days, 4 days, 5 days, 6 days, 7 days, or I do not know.
3. Duration of moderate to vigorous physical activities: In the days when your child practiced physical activities to strengthen his/her bones and muscles (e.g., rope jumping, climbing an elevated object, running), how much time does your child approximately spend per day doing this activity? Response options: None, approximately less than 30 min, approximately more than 30 min, approximately 60 min or more, or I do not know.

### 2.3. Data Analysis

Data were analyzed using SPSS (version 30). Descriptive statistics (frequency, percentage, mean, standard deviation, minimum, and maximum) were computed for the demographic variables, physical activity items, and QoL domain scores. Independent samples *t*-tests were performed to compare the three physical activity variables between the children with CP and those without CP. Levene’s Test for Equality of Variances was used to determine whether the assumption of homogeneity of the variance was met. Group statistics (mean, standard deviation, and standard error) for each variable were computed and are presented along with *t*-statistics, degrees of freedom, two-tailed significance values, mean differences, and 95% confidence intervals for the differences. Two sets of regression analyses were conducted with physical activity as the dependent variable for the children with CP. A simple linear regression was performed with the physical activity score as a predictor of general QoL. A multiple regression model was used to determine whether physical activity predicted the seven individual QoL domain scores. Model summary statistics (R, R^2^, adjusted R^2^, and the standard error of estimate) were obtained for each model, and ANOVA was used to test the overall significance of the models. Unstandardized regression coefficients (B), standard errors, standardized regression coefficients (Beta), *t*-values, and significance levels for each predictor were also obtained. Independent sample *t*-tests were used to compare general QoL and the seven QoL domain scores between the ambulant and non-ambulant groups of children with CP. For each variable, the group means, standard deviations, standard errors, *t*-values, degrees of freedom, *p*-values, and 95% confidence intervals for the mean differences were calculated.

## 3. Results

### 3.1. Demographic Characteristics

A total of 114 participants completed the survey instrument: 55 children with CP [mean age = 8.1 years (SD 2.26)] and 59 TD children [mean age = 8.2 years (SD 2.26)]. Out of 114, 32 children were excluded. Thirteen children with CP and 12 TD were excluded because they did not meet the age criteria. Additionally, seven children with TD were excluded because they reported a form of disability or pain that affected their physical activity. Therefore, 82 participants were included in the analysis: 42 children with CP and 40 typically developing children (8.29 ± 1.79 years; 8.35 ± 1.76 years). Participants’ characteristics are presented in Table 1.

### 3.2. Days of Physical Activity of Children with Cerebral Palsy and Typically Developing Children

**Figure 1:**
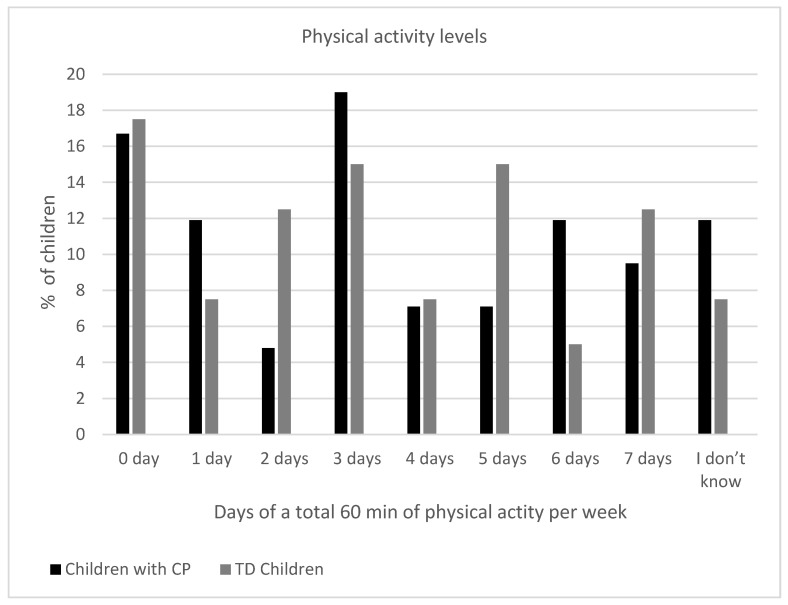
Physical activity levels among children with cerebral palsy and typically developing children in the last seven days.

**Figure 2:**
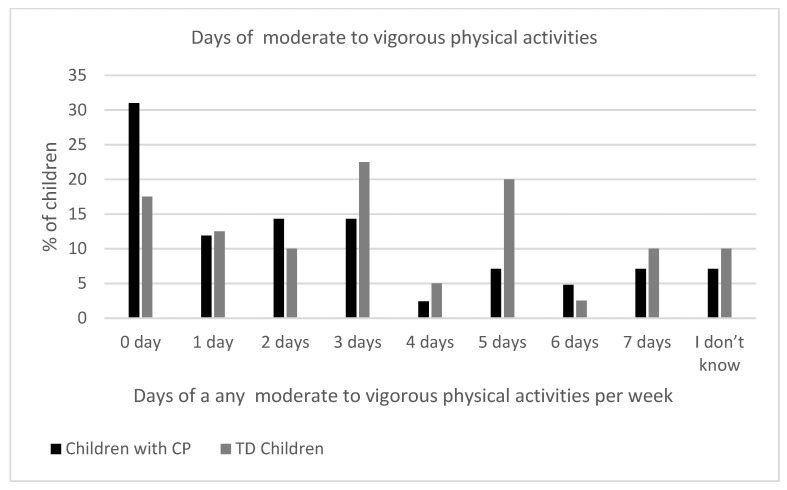
Days of moderate to vigorous physical activities among children with cerebral palsy and typically developing children in the last seven days.

**Figure 3:**
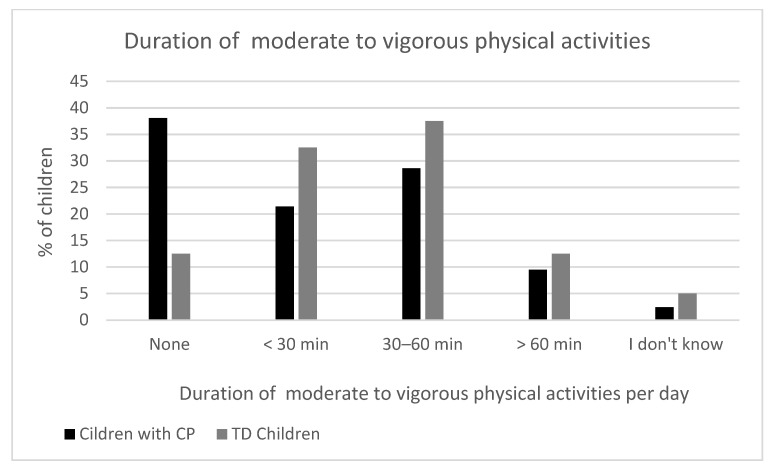
Duration of moderate to vigorous physical activities among children with cerebral palsy and typically developing children in the last seven days.

There was no statistically significant difference between the groups in the number of days they participated in 60 min of physical activity (*p* = 0.923). Activity levels were generally low in both groups, with the most common response being 0 or 3 days per week. The physical activity levels of the children with CP and the typically developing controls are shown in Figure 1. Children with CP appeared to engage less often in moderate to vigorous physical activities compared to TD children, although the difference did not reach statistical significance (*p* = 0.111). For example, 31% of children with CP reported 0 days of moderate to vigorous physical activities, compared to only 17.5% of the TD children (Figure 2). A marginally significant difference was found in the number of minutes spent on moderate to vigorous physical activities between the two groups (*p* = 0.054). Notably, 38.1% of the children with CP engaged in 0 min of moderate to vigorous physical activities compared to only 12.5% of the TD children, suggesting a trend toward lower intensity exercise in the CP group (Figure 3). Supplementary Table S1 shows the comparison of physical activity levels between children with CP and TD controls.

### 3.3. Quality of Life

The descriptive statistics of QoL among the children with CP are presented in Table 2. The QoL domains with the highest (better) scores were social well-being and acceptance [Mean = 71.51 (SD = 26.29; range = 5.68–100)] and emotional well-being and self-esteem [Mean = 66.82 (SD = 19.59; range = 0.00–87.50)]. The QoL domains with the lowest (worst) scores were the access to service [Mean = 37.7 (SD = 18.26; range = 0.00–77.88)] and the pain and effect of disability [Mean = 41.74 (SD = 19.49; range: 0.00–75.00)].

The majority of children with CP were classified as having moderate to severe motor limitations, with 33.3% transported in a manual wheelchair and 21.4% using self-powered mobility, while 45.3% children were ambulatory. The only significant difference between these groups was observed in the “feelings about functioning” domain. Ambulant children with CP (GMFCS I-III) had better parent-reported QoL than the non-ambulant children (GMFCS IV-V) had in the domains of feelings about functioning (Table 3).

### 3.4. The Association Between Physical Activity and Quality of Life in Children with Cerebral Palsy

Physical activity significantly predicted general QoL scores in children with CP (*p* = 0.003), explaining 19.5% of the variance. For each day of physical activity, general QoL increased by approximately 4.94 scores. Days of physical activity predict social well-being and acceptance, feelings about functioning, participation, physical health, emotional well-being, and Self-esteem scores. Physical activity did not significantly predict access to services (*p* = 0.494), which suggests that a child’s level of physical activity is independent of their perceived access to therapy, medical support, and educational services. Similarly, physical activity did not significantly predict pain, disability (*p* = 0.131), or family health (*p* = 0.356), as shown in Table 4.

## 4. Discussion

This study examined physical activity levels and the QoL of children with CP and their TD peers. The study further explored the relationships between the QoL domains and physical activity within the CP group. Comparisons were made between ambulant (GMFCS I–III) and non-ambulant (GMFCS IV–V) children with CP to determine whether motor function influences various QoL domains. Our results indicate that children with CP engage in similar levels of physical activity as TD children do. However, both groups exhibited low physical activity levels, with the majority participating in 0–3 days of activity per week. The TD children in this study fell short of meeting the international guidelines for the physical activity of children which agreed with a previous review that reported a low global prevalence of physical activity among children and adolescents [24]. Similarly, we found that children with CP are not meeting the physical activity recommendations, which aligns with the current research literature. This is especially true in the Saudi Arabia context, as it is noted that the physical activity levels of children in Saudi Arabia are generally low, and there is a notable lack of awareness regarding the recommended amount of physical activity. Moreover, several factors may contribute to the low number of physical activity days observed in the two groups in this study. First, this may indicate that children with CP are not necessarily excluded from participating in regular activities, which could be a positive sign of inclusive practices and accessible opportunities, however awareness in children physical activity, in general, is lacking. Second, the use of a parent-reported tool to measure physical activity might reflect the perceived frequency rather than the objective intensity, duration, or exertion, and whether parents classify therapy sessions, slow walking, or passive play as physical activity.

The findings of low physical activity in groups of children with and without CP highlight the need for targeted interventions and support to enhance children’s participation in physical activities, including those with CP [25,26,27].

An increase in the number of days of physical activity was positively associated with the emotional and social aspects of the quality of life of the children with CP in this study. These findings align with a study that showed that physical activity was associated with higher QoL scores in domains related to social aspects of life, although no effect was found on emotional scores [10]. In contrast, a study on ambulatory school-age children with cerebral palsy found that physical activity energy expenditures predicted the physical, emotional, and social aspects of QoL in children with CP [28].

The motor function of the children with CP did not explain aspects of QoL, except for feelings about one’s own functioning. Similarly, previous studies reported that ambulant children with CP (GMFCS I–III) had better parent-reported QoL than did the non-ambulant children (GMFCS IV–V) in the domain of feelings about functioning [29]; yet, investigating QoL of children with CP showed that motor function explains some aspects of QoL, including the access to services domain [30]. This difference suggests that mobility may significantly influence how children with CP perceive their ability to function independently. However, general QoL and other domains (social well-being, participation, emotional well-being, access to services, pain, effects of disability, and family health) did not show any significant differences between the two groups in our study.

These findings show that, from the perspective of parents, certain factors (e.g., home adaptation, supportive laws and policies, and family-centered rehabilitation services may enhance the QoL of children with CP. Interventions that address modifiable environmental features, improve children’s fine motor abilities and interaction skills, and help families manage behavioral difficulties, may optimize the QoL of school-age children with CP [31].

Rehabilitation professionals’ attitude and access to rehabilitation services play a pivotal role in influencing both the child’s and the family’s journey. They can mitigate QoL-related challenges in early intervention settings by prioritizing parents’ perceptions and employing QoL assessments [32]. This approach not only fosters the well-being and self-determination in children with CP but also ensures that healthcare providers are well-informed about available governmental and non-governmental support systems in Saudi Arabia. It is noteworthy that the present study found that physical activity levels might not exert a strong influence on the remaining aspects of QoL, thereby warranting further investigation into those specific domains to fully understand the complexities at play to enhance the overall QoL of children with CP.

Our results indicate that approximately 26% of children with CP are not attending school. This observation aligns with results of several studies conducted in Saudi Arabia that have reported lower rates of school attendance among children with CP [13,30]. Conducting more comprehensive studies to investigate the underlying reasons for the lower rates of school attendance among children with CP is essential. Understanding these factors is crucial for developing effective interventions. One potential contributing factor identified in our study is that the majority of children with CP (71%) reside outside of Riyadh, the capital city. This geographical disparity suggests that transportation challenges hinder children’s access to educational facilities, particularly in rural areas, compared to their urban counterparts. The previous factor of children with CP not attending school can play a critical role in not only low physical activity, but it can also influence the psychological and social results that the parents report.

The findings of this study have several important implications. Our results highlight the critical need for evidence-based practice guidelines on physical activity in Saudi Arabia, which should provide unified recommendations and information for children and adolescents with CP and their families. Such guidelines should influence the QoL of children and their families, thereby assisting healthcare providers to develop valid evidence-based recommendations. The significant association between QoL (i.e., the domain of feelings about functioning and physical activity) indicates the need for interventions that boost children’s self-confidence and functional perceptions. In addition, low physical activity was reported in both children with CP and TD children, which suggests the need for more awareness of physical activity guidelines to the general population. And emphasizing that long-term consequences of reduced habitual physical activity and increased sedentary behavior include a greater risk of developing chronic diseases.

Therapy and behavior change interventions may increase physical activity participation among children with CP, which might lead to an increase in physical activities essential for health and social integration [26,33]. Finally, lack of significant differences in most QoL domains between ambulant and non-ambulant children indicates that factors beyond motor function (e.g., psychosocial support, adaptive equipment, or environmental modifications) might mitigate the effect(s) of mobility limitations on overall QoL. Future studies should explore these compensatory factors in greater detail and examine longitudinal relationships among physical activity, QoL, and motor function. The QoL assessments of children with CP should extend beyond functional abilities in order to include less obvious, but critical, psychological and social issues. Future studies should examine how different socioeconomic factors affect the QoL of children with CP. Furthermore, we need further studies to investigate QoL and its association with physical activity in adolescents and adults with CP using a similar methodology.

This study adds to our understanding and improves our current knowledge of the relationship between physical activity and QoL in children with CP in general, and in Saudi Arabia. Our study used validated and well-established self-report assessments that have been translated into Arabic with reliable methods. The use of multiple self-report assessments (GMFCS, CP QoL, and physical activity) provided a more comprehensive overview of the QoL of children with CP.

Despite our study’s strengths, it has some limitations. This study did not use objective measures of physical activity, such as an accelerometer, which can provide measures of children’s physical activity levels that are more accurate. Future research is needed to investigate the agreement between objective physical activity measures and parents’ self-reports in children with CP. The relatively small sample size restricts the generalizability of our findings. Our results were based on parents’ self-reported assessments, and it is essential to note that disparities exist between parents’ and children’s self-reports, particularly in the physiological domain [4]. It has been reported that children with CP usually rate their QoL in both the emotional and social domains as equal to that of their TD peers [4]. Moreover, parents of children with severe CP impairments (GMFCS levels IV and V) often report better QoL in the psychosocial domains compared with parents of children with mild impairments [4]. It is critical to develop evidence-based physical activity guidelines to provide recommendations for children and adolescents with CP. Therefore, future studies should include a larger sample size, use objective measures, examine socioeconomic factors, and conduct multiple follow-ups to ensure the generalizability of the findings, thereby validating the current results.

## 5. Conclusions

Our study highlights the potential benefits of physical activity for children with CP, particularly for enhancing general QoL, social well-being, acceptance, feelings about functioning, participation, and emotional well-being and self-esteem. Although our findings show a strong association between physical activity levels and these aspects of QoL, evidence of the broader effect(s) on other domains is limited. Moreover, differences in overall QoL based on the GMFCS level are minimal. Nevertheless, self-perceptions of functioning differ significantly, thereby highlighting a potential target for therapeutic interventions. Addressing these perceptions may not only improve levels of physical activity but also contribute to a better overall QoL for children with CP. Future research should investigate this relationship in greater depth by considering a broader range of physical activities and employing more sensitive measures of physical activity.

## Figures and Tables

**Table 1:** Characteristics of children with cerebral palsy and typically developing controls.

	Children with CP (*n* = 42)	TD Children (*n* = 40)
**Age (years), mean (SD)**	8.29 ± 1.79	8.35 ± 1.76
**Sex**		
**Male, *n* (%)**	28 (66.7%)	14 (35%)
**Female, *n* (%)**	14 (33.3%)	26 (65.5%)
**Residence**		
**Riyadh, *n* (%)**	12 (28.6%)	33 (82.5%)
**Outside Riyadh, *n* (%)**	30 (71.4%)	7 (17.5%)
**Attending school**	31 (74%)	40 (100%)
**Caregiver education**		
**High school or less, *n* (%)**	10 (23.8%)	12 (30%)
**Diploma, *n* (%)**	6 (14.3%)	4 (10%)
**Bachelor, *n* (%)**	24 (57.1%)	19 (47.5%)
**Postgraduate studies, *n* (%)**	2 (4.8%)	5 (12.5%)
**GMFCS level**		
**Level I, *n* (%)**	2 (4.8%)	-
**Level II, *n* (%)**	8 (19%)	-
**Level III, *n* (%)**	9 (21.4%)	-
**Level IV, *n* (%)**	9 (21.4%)	-
**Level V, *n* (%)**	14 (33.3%)	-

CP, Cerebral palsy; GMFCS, Gross Motor Function Classification System; *n*, number; TD, typically developing.

**Table 2:** Quality of life domains scores in children with cerebral palsy.

Quality of Life Domain	*n*	Min	Maxi	Mean	Std. Deviation
Social well-being and acceptance	42	5.68	100.00	71.5097	26.29243
Feelings about functioning	42	8.33	96.88	56.6716	25.87401
Participation and physical health	42	2.27	100.00	61.0119	26.31170
Emotional well-being and self-esteem	42	0.00	87.50	66.8155	19.59421
Access to services	42	0.00	77.88	37.7060	18.26434
Pain and effect(s) of disability	42	0.00	75.00	41.7426	19.48737
Family health	42	15.00	100.00	63.5714	25.54971

Max, maximum score provided by caregivers; Min, minimum score provided by caregivers per domain; *n*, number. QoL domains scores range from 0 to 100 (maximum).

**Table 3:** Quality of life of ambulatory and non-ambulatory children with cerebral palsy.

Quality of Life Domain	Ambulation Status (GMFCS Level)	*n*	Mean	Std. Deviation	Std. Error Mean
General QoL	Ambulant (I–III)	19	55.39	16.14	3.70
	Non-Ambulant (IV–V)	23	52.46	18.21	3.79
Social well-being and acceptance	Ambulant (I–III)	19	71.11	24.44	5.60
	Non-Ambulant (IV–V)	23	71.84	28.27	5.89
Feelings about functioning	Ambulant (I–III)	19	65.57	24.25	5.56
	Non-Ambulant (IV–V)	23	49.32	25.33	5.28
Participation and physical health	Ambulant (I–III)	19	64.35	22.14	5.08
	Non-Ambulant (IV–V)	23	58.25	29.52	6.15
Emotional well-being and self-esteem	Ambulant (I–III)	19	66.34	21.09	4.84
	Non-Ambulant (IV–V)	23	67.21	18.74	3.91
Access to services	Ambulant (I–III)	19	35.83	15.89	3.64
	Non-Ambulant (IV–V)	23	39.25	20.24	4.22
Pain and effect(s) of disability	Ambulant (I–III)	19	41.81	19.65	4.51
	Non-Ambulant (IV–V)	23	41.69	19.79	4.13
Family health	Ambulant (I–III)	19	63.68	24.30	5.57
	Non-Ambulant (IV–V)	23	63.47	27.08	5.65

GMFCS, Gross Motor Function Classification System; Mean, mean of QoL scores provided by parents per domain and ranging from 0 (worst score) to 100 (best score); *n*, number.

**Table 4:** Association between physical activity and quality of life domains among children with cerebral palsy.

Regression Coefficients						
Model		Unstandardized Coefficients		Standardized Coefficients	t	Sig.
		B	Std. Error	Beta		
General QoL	(Constant)	43.393	4.116		10.542	0.000
	PA	4.941	1.588	0.441	3.111	0.003 *
Social well-being and acceptance	(Constant)	56.260	6.370		8.832	0.000
	PA	7.251	2.458	0.423	2.950	0.005 *
Feelings about functioning	(Constant)	39.124	6.013		6.506	0.000
	PA	8.343	2.320	0.494	3.596	0.001 *
Participation and physical health	(Constant)	45.613	6.362		7.169	0.000
	PA	7.322	2.455	0.427	2.983	0.005 *
Emotional well-being and Self-esteem	(Constant)	57.020	4.878		11.689	0.000
	PA	4.658	1.882	0.364	2.474	0.018 *
Access to services	(Constant)	34.989	4.854		7.208	0.000
	PA	1.292	1.873	0.108	0.690	0.494
Pain and effect(s) of disability	(Constant)	35.404	5.061		6.995	0.000
	PA	3.014	1.953	0.237	1.543	0.131
Family health	(Constant)	58.451	6.757		8.650	0.000
	PA	2.435	2.607	0.146	0.934	0.356

PA, Physical activity; Std, standard; * indicate *p*-value < 0.05.

## Data Availability

The data that support the findings of this study are available from the corresponding author upon request.

## Supplementary Materials

The following supporting information can be downloaded at: https://www.mdpi.com/article/10.3390/healthcare13172166/s1, Table S1: Comparison of physical activity levels between children with CP and TD controls.

## Abbreviations

CP	Cerebral Palsy
CP-QoL	Quality of Life Questionnaire for Parents of Children with Cerebral Palsy
CP-QoL-Child	Quality of Life Questionnaire for Children Aged 4–12 Years with Cerebral Palsy
GMFCS (E&R)	Gross Motor Function Classification System (Expanded & Revised)
