# Supplementary material for: Quality of Life of Children with Cerebral Palsy and Its Association with Their Physical Activity Levels: A Cross-Sectional Study
Source: Healthcare (Basel). 2025 Aug 30;13(17):2166. doi: 10.3390/healthcare13172166 (PMC12428702; doi:10.3390/healthcare13172166)
Supplement: Supplementary file 1 [file healthcare-13-02166-s001.zip › healthcare-3817364-supplementary.pdf]

Supplementary Table 1: Comparison of physical activity levels between children with CP and TD controls.

	Children with CP n=42	TD Children n=40	p values
In the last 7 days, how many days did your child participate in 60 minutes of physical activity per day?			0.923
0 day	7(16.7%)	7(17.5%)	
1 day	5(11.9%)	3(7.5%)	
2 days	2(4.8%)	5(12.5%)	
3 days	8(19%)	6(15%)	
4 days	3(7.1%)	3(7.5%)	
5 days	3(7.1%)	6(15%)	
6 days	5(11.9%)	2(5%)	
7 days	4(9.5%)	5(12.5%)	
I do not know	5(11.9%)	3(7.5%)	
In the last 7 days, how many days did your child participate in strengthening exercises (high-intensity) per day?			0.111
0 day	13(31%)	7(17.5%)	
1 day	5(11.9%)	5(12.5%)	
2 days	6(14.3%)	4(10%)	
3 days	6(14.3%)	9(22.5%)	
4 days	1(2.4%)	2(5%)	
5 days	3(7.1%)	8(20%)	
6 days	2(4.8%)	1(2.5%)	
7 days	3(7.1%)	4(10%)	
I do not know	3(7.1%)	4(10%)	
In the last 7 days, in the days your child participated in strengthening exercises (high-intensity), how many minutes of strengthening exercises did your child perform?			0.054*
None	16(38.1%)	5(12.5%)	
Less than 30 min	9(21.4%)	13(32.5%)	
Between 30 and 60 min	12(28.6%)	15(37.5%)	
More than 60 min	4(9.5%)	5(12.5%)	

I do not know	1(2.4%)	2(5%)

CP, Cerebral palsy; n, Number; PA, Physical activity; TD, Typically developing

*p-value significant <0.05, independent sample t-test
